# Understanding the Mechanisms of Proteinuria: Therapeutic Implications

**DOI:** 10.1155/2012/546039

**Published:** 2012-07-04

**Authors:** Jorge E. Toblli, P. Bevione, F. Di Gennaro, L. Madalena, G. Cao, M. Angerosa

**Affiliations:** ^1^Laboratorio de Medicina Experimental, Hospital Alemán, Facultad de Medicina, University of Buenos Aires, Avenida Pueyrredon 1640, 1118 Buenos Aires, Argentina; ^2^Servicio de Nefrología, Hospital Alemán, 1118 Buenos Aires, Argentina; ^3^Departamento de Medicina Interna, Hospital Alemán, 1118 Buenos Aires, Argentina; ^4^Departamento de Bioquímica Clínica, Facultad de Farmacia y Bioquímica, Universidad de Buenos Aires, 1113 Buenos Aires, Argentina; ^5^Hospital de Clinicas “José de San Martín”, Universidad de Buenos Aires, 1120 Buenos Aires, Argentina

## Abstract

A large body of evidence indicates that proteinuria is a strong predictor of morbidity, a cause of inflammation, oxidative stress and progression of chronic kidney disease, and development of cardiovascular disease. The processes that lead to proteinuria are complex and involve factors such as glomerular hemodynamic, tubular absorption, and diffusion gradients. Alterations in various different molecular pathways and interactions may lead to the identical clinical end points of proteinuria and chronic kidney disease. Glomerular diseases include a wide range of immune and nonimmune insults that may target and thus damage some components of the glomerular filtration barrier. In many of these conditions, the renal visceral epithelial cell (podocyte) responds to injury along defined pathways, which may explain the resultant clinical and histological changes. The recent discovery of the molecular components of the slit diaphragm, specialized structure of podocyte-podocyte interaction, has been a major breakthrough in understanding the crucial role of the epithelial layer of the glomerular barrier and the pathogenesis of proteinuria. This paper provides an overview and update on the structure and function of the glomerular filtration barrier and the pathogenesis of proteinuria, highlighting the role of the podocyte in this setting. In addition, current antiproteinuric therapeutic approaches are briefly commented.

## 1. Introduction 

Proteinuria is considered a major healthcare problem that affects several hundred million people worldwide. In addition, proteinuria is a sensitive marker for progressive renal dysfunction and it is considered an independent risk factor for cardiovascular (CV) morbidity and mortality [1]. 

Furthermore, it is widely accepted that microalbuminuria (albumin urinary excretion of 30 mg–300 mg/day) is the earliest clue about the renal involvement of diabetes, obesity, and the metabolic syndrome. Interestingly, while microalbuminuria is more predictive of reaching CV end points than kidney end points, macroalbuminuria (total protein urinary excretion >500 mg/day) has been demonstrated to be more associated with reaching kidney end points [2].

However, microalbuminuria can often progress to overt proteinuria leading 10–50% of the patients to end-stage kidney disease development, ultimately requiring dialysis or transplantation. Of similar importance is the observation that even levels of albumin under the microalbuminuria threshold (so-called ‘‘high normal”) are associated with an increased risk for CV outcomes [3]. Therefore, a reduction or prevention of protein urine excretion is highly desirable. 

It is worth remembering that the current staging system for chronic kidney disease (CKD) is based primarily on estimated glomerular filtration rate (eGFR) with lower eGFR associated with a higher risk of adverse outcomes. Moreover, the risks of mortality, myocardial infarction, and progression to chronic renal failure associated with a given level of eGFR are independently increased in patients with higher levels of proteinuria. In fact, patients with heavy proteinuria but without overtly abnormal eGFR appear to have worse clinical outcomes than those with moderately reduced eGFR but without proteinuria [4]. Although proteinuria is also associated with poor renal outcomes, the current guidelines have been criticized because they do not incorporate information about the presence and severity of proteinuria, an important marker of CKD that is associated with adverse outcomes [5–8]. 

As the measurement and sampling procedures for proteinuria assessment have not been standardized yet, it is of clinical importance to take into account different types of urinary proteins, albumins, laboratory techniques, and urine sampling methods in order to have the best approach for an individual patient.

Total urinary protein can be assessed using dipstick, precipitation, and electrophoresis methods. Urinary albumin, the predominant urinary protein in most proteinuric renal diseases, can be evaluated using an albumin-specific dipstick, immunochemical techniques, and size-exclusion high-performance liquid chromatography. In addition, urine albumin may be immune-reactive, immune-unreactive, fragmented, and biochemically modified, and assorted laboratory techniques have variable abilities to detect different types of albumin. 

Urine specimen for proteinuria assessment can be obtained either from a timed collection or a spot urine sample. Nevertheless, currently spot urine protein- or albumin-to-creatinine ratios are preferred to a 24-hour urine sample in routine practice. Moreover, urinary ratios are also helpful in monitoring changes in the degree of proteinuria in CKD patients [4]. Whereas the assessment of albuminuria in patients with diabetic nephropathy is of paramount importance, proteinuria and albuminuria tests both have a role in nondiabetic kidney disease and in general population screening [9]. 

It is widely accepted that proteinuric nephropathies seem to progress independently of their initial aggression type leading to irreversible parenchyma damage and end-stage renal disease if otherwise unattended. 

The molecular mechanisms that lead to proteinuria and podocyte effacement have been poorly understood for a long time; consequently, targeted therapies have been lacking. Fortunately, an interesting body of data has emerged in this field in the last few years [10]. The discovery of podocyte gene defects that underlie some hereditary proteinuric syndromes has changed our understanding of the relative contributions of the components of the glomerular filter. Additionally, the pathogenic pathways activated in podocytes during proteinuria have been identified. Based on this scenario, therapeutic strategies for controlling urinary protein excretion, which may contribute to delaying or stopping GFR loss, acquire clinical relevance.

## 2. Physiopathology of Proteinuria: Mechanism and Progression of Renal Disease

**Figure 1:**
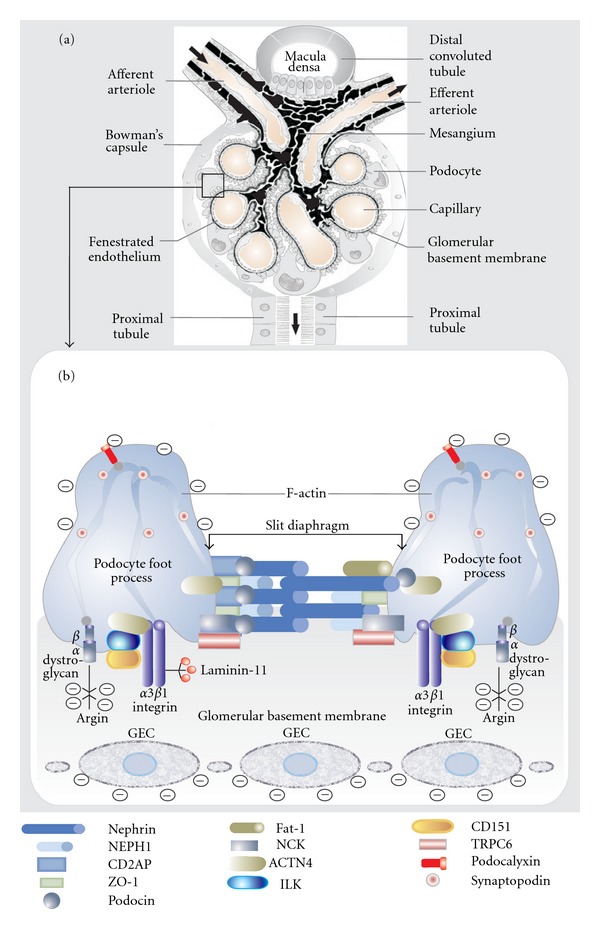
Schematic representation of glomerular filtration barrier components. (a) Illustration of all participant structures in the normal glomerular filtration process. The rectangle indicates the tight relationship between the glomerular capillary wall, glomerular basement membrane, and glomerular visceral epithelial cell (podocyte) which is detailed in the lower panel. (b) Molecular structures of the podocyte and slit diaphragm. Abbreviations: ACTN4: *α*-actinin-4; CD2AP: CD2-associated protein; GEC: glomerular endothelial cell; ILK: integrin-linked kinase; ZO-1: tight junction protein ZO-1; CD151: tetraspanin CD151; TRPC6: transient receptor potential cation channel 6; NCK: protein adaptor NCK.

The microscopy architecture of the “glomerular filtration barrier” is constituted by three different layers, the glomerular endothelial cell, the glomerular basement membrane, and the visceral epithelial cell or podocyte. Although all of them are important to preserve normal glomerular function, the podocyte, the most differentiated cell type in the glomerulus, seems to be the essential part of the filtration unit (Figure 1). 

### 2.1. Glomerular Endothelial Cell (GEC)

One of the characteristics of GEC is the presence of numerous fenestrations. These are round or ovoid transcellular holes through the most attenuated part of the GEC cytoplasm. Since these openings are substantially large in relation to the side of the albumin molecule, a considerable amount of albumin might pass through them. Nevertheless, GEC has a cell-surface layer, namely, “glycocalyx,” which in normal conditions impedes the leakage of free albumin as well as other proteins. This glycocalyx is principally composed of proteoglycans and sialoproteins [11]. Biophysical models indicate that fenestral glycocalyx contributes to 50% of the overall hydraulic resistance of the glomerular filtration barrier [12]. Therefore, changes in the amount or composition of glycocalyx within the fenestrae would also have significant effects on GFR. In agreement with this concept, some experimental studies have reported that defects in GEC glycocalyx were associated with proteinuria, which highlights the importance of this complex structure [13, 14]. 

Several lines of evidence showed that the vascular endothelial growth factor (VEGF), the best characterized angiogenic/vasculogenic factor, is a critical “cross-talk” protein among the components of the glomerular filtration barrier [15]. VEGF, mainly synthesized by podocytes, is required for normal GEC function as clinical and experimental data have demonstrated [16–19]. However, contradictory data emerge from animal models or human kidney biopsies of various glomerulopathies. In minimal change nephropathy (MCN), VEGF, and its receptors were, as well as in diabetic nephropathy, upregulated and correlated with the severity of proteinuria [20, 21]. In patients with MCN and nephrotic syndrome, the urinary VEGF levels are increased and positively correlated with the degree of proteinuria [22]. 

In addition, renal biopsies from patients with membranous glomerulonephritis (MGN), membranoproliferative glomerulonephritis (MPGN), endocapillary nephritis, and crescentic nephritis presented markedly increased VEGF protein in podocytes [23]. 

Therefore, the role and/or interaction of podocyte-derived VEGF in glomerular health and disease still remain as open debate.

### 2.2. Glomerular Basement Membrane (GBM)

For many years, the GBM has been considered to have a key role in the glomerular filtration of macromolecules. The essential component in the GBM is type IV collagen, which is early secreted by GEC during fetal development (*α*1*α*2*α*1), and is later replaced by the more robust collagen network constituted primarily of *α*3*α*4*α*5 heterotrimers secreted by the podocyte [24]. The GBM also contains laminin, nidogen, and sulfated proteoglycan, components found in all basement membranes, but for some of these classes, the specific isoforms present in the GBM are very different from those found in other basement membranes. Most of these proteins are produced by podocytes and in less proportion by GECs [25]. 

Type IV collagen is organized as a crosslinked network of triple-helical molecules that primarily provide structural support to the glomerular capillary wall and make little contribution to the size-selectivity or charge-selectivity of the glomerular filter. Emphasizing this concept, mutations in the genes that encode GBM type IV collagens result in collagen *α*5 (IV) alteration, this leading to the Alport syndrome, where hematuria in conjunction with moderate proteinuria is present [26]. 

The proteoglycans are heterogeneous molecules composed of a core protein with covalently attached glycosaminoglycan side chains (GAGs). The two best studied proteoglycans in the GBM are perlecan and agrin. They have heparan sulfate as a GAG side chain that provides for at least part of the GBM charge selectivity against the passage of negatively charged molecules into urine. Interestingly, although intravenous administration of heparanases results in increased glomerular permeability to ferritin, which suggests a competent role of heparan sulfate proteoglycans in the filtration barrier, other experimental studies have minimized the actual protein loss [27, 28]. 

Laminins are heterotrimeric proteins that self-assemble into a network in basement membranes. Laminin-11 (*α*5, *β*2, and *γ*1 chains) is found in mature GBM and connects to collagen IV through nidogen and entactin. The autosomal recessive mutations in laminin *β*2 gene result in the Pierson syndrome, which is characterized as a congenital nephrotic syndrome accompanied by ocular and neurological defects. Most of the patients with the Pierson syndrome who are diagnosed with nephrotic-range proteinuria rapidly develop ESRD and die in the perinatal period [29]. 

### 2.3. Visceral Epithelial Cell (Podocyte)

**Figure 2:**
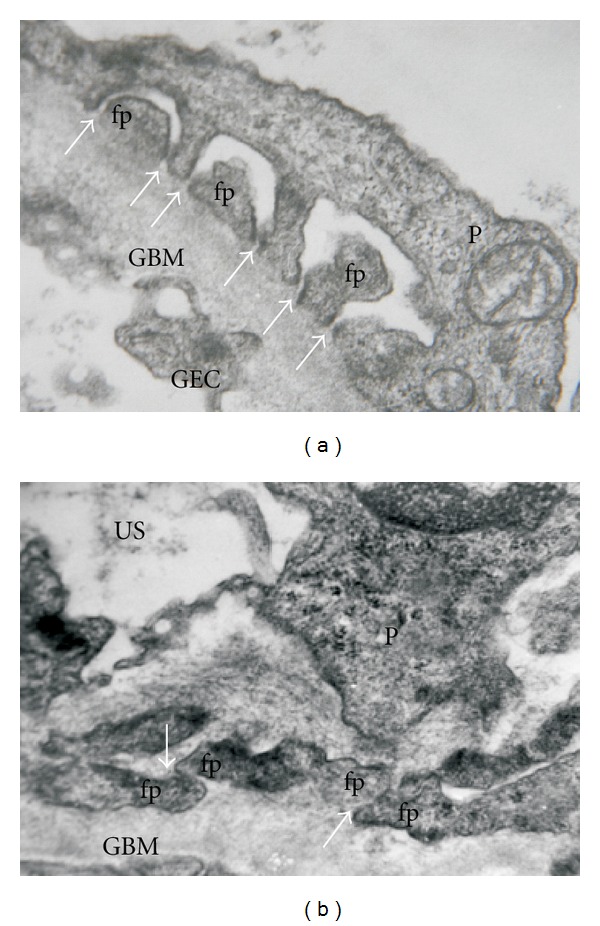
Glomerular filtration barrier in normal and pathological conditions. (a) A cross-section of a normal human glomerular capillary. Arrows indicate the foot processes with interconnecting ultrathin slit diaphragms. (b) Significant changes in the glomerular filtration barrier in a patient with heavy proteinuria due to a primary glomerulopathy. Note the foot process effacement with alteration in slit diaphragm (arrows), which results in a disarrangement of the functional filter structure. Abbreviations: fp: foot process; GBM: glomerular basement membrane; P: podocyte; GEC: glomerular endotelial cell; US: urinary space. (Transmission electron microscopy. Uranyl acetate-lead citrate original magnification ×40,000.)

Podocytes are the largest cells in the glomerulus; they have long cytoplasm processes which extend from the main cell body and divide into individual foot processes (pedicels) that attach the cell to the GBM (Figure 2). A considerable number of microtubules and microfilaments are present in the cytosol of podocytes, together with actin filaments, which are particularly abundant in the foot processes (Figure 1). In normal conditions, the distance between adjacent foot processes near the GBM varies from 25 nm to 60 nm. This gap is bridged by a thin membrane coined as “slit diaphragm” (Figure 1). 

Podocytes are anchored to components of the underlying GBM via transmembrane cell receptors such as *αβ*-dystroglycan and integrins, the *αβ* heterodimeric proteins that are generally responsible for connecting epithelial cells to basement membranes. *α*3*β*1 integrin is the most abundant isoform present in podocytes and is localized exclusively to the basal membrane, linked to the actin cytoskeleton (Figure 1). Furthermore, the *α*3 chain is necessary for the development of the glomerular capillary tuft. Mice in which the integrin *α*3 gene is inactivated during podocyte development show massive proteinuria within the first week of life and electron microscopy shows complete foot process effacement and widespread lamination with protrusions of the GBM [30]. The mechanism which explains the presence of proteinuria in integrin-deficient mice is not totally clear. Nevertheless, since *α*3*β*1 integrin is a major receptor for laminin, the disruption of the integrin-laminin complex could result in the weakening of the podocyte-GBM interaction and progressive detachment of podocytes, which is associated with proteinuria.

Integrin-linked kinase (ILK) seems to have an essential role in the glomerular filtration barrier. Podocyte ILK was found to be upregulated in human proteinuric glomerular diseases. Additionally, ectopic expression of ILK in podocytes decreased levels of the epithelial markers nephrin and ZO-1, induced mesenchymal markers such as desmin, fibronectin, matrix metalloproteinase-9, and alpha-smooth muscle actin, promoted cell migration, and increased the paracellular albumin flux across podocyte monolayers [31–33]. Another participant in the podocyte-GBM interface is tetraspanin CD151, which has a strong lateral interaction with integrin *α*3*β*1. This interaction is important for the adhesion to the GBM as CD151-knockout mice develop proteinuria within a few weeks of birth and electron microscopic examination reveals the presence of lamination and spikes in the GBM as well as focal foot process effacement, though with less severity than that seen in integrin *α*3 knockout mice [30]. It is worth mentioning that a contractile structure, composed of actin, myosin, *α*-actinin-4, vinculin and talin, is present in the podocyte foot processes. This group of proteins is connected to the GBM at focal contacts by the *α*3*β*1 integrin complex [34]. 

Podocytes are also covered by glycocalyx, principally podocalyxin, a sialomucin closely related to CD34 and endoglycan. Through interactions with several intracellular proteins and at least one extracellular ligand, podocalyxin regulates both adhesion and cell morphology. In the developing kidney, podocalyxin plays an essential role in the formation and maintenance of podocyte foot processes, and its absence results in perinatal lethality [35]. In puromycin aminonucleoside nephrosis and protamine sulfate perfusion studies, podocalyxin's negative charge is neutralized, foot process architecture is disrupted, and slit diaphragms are displaced or completely replaced by leaky, discontinuous junctions [36]. 

The slit diaphragm is one of the major impediments to protein permeability across the glomerular filtration barrier. Consequently, alterations in the cytoskeletal architecture and/or expression of slit diaphragm proteins can be present in most nephrotic disorders. In recent years, the discovery of the molecular basis of the regulation and function of the slit diaphragm structure and its relation with genetic forms of nephrotic syndrome has placed the podocyte as the target cell linked to the development of proteinuria. Nonetheless, from the clinical point of view, it is relevant to distinguish the proteinuria in the case of genetic mutations of slit diaphragm proteins from that which develops in the scenario of arterial hypertension, diabetes, and progressive CKD. In these particular conditions, a GEC alteration with loss of charge selectivity of glycocalyx is probably the first event. This exposes the podocytes to the deleterious effects of albumin and other macromolecules. It is worth emphasizing that in metabolic diseases such as diabetes, albumin may undergo glycation and nitration, which cause substantial structural and functional modifications in its protein configuration [37]. Consequently, the continuous exposition to modified albumin may directly lead to alterations in podocyte function and the disarrangement of slit diaphragm structure. 

Both, CD2AP (CD2-associated protein) and NCK (Figure 1) are linker proteins which connect the slit diaphragm to the actin cytoskeleton of podocytes. CD2AP directly interacts with actin (F-actin) and synaptopodin, an actin-bundling protein. Furthermore, CD2AP also interacts with nephrin and podocin in the slit diaphragm (Figure 1). Mutations in CD2AP may cause focal segmental glomerulosclerosis (FSGS) depending on their severity [38]. The interaction with CD2AP might predominate in a steady-state situation, whereas the interaction with NCK proteins could be important during injury and development.

Using diverse techniques as fractionation, immunofluorescence, and immunoelectron microscopy, tight junction proteins such as junction adhesion molecule A, occluding, and zone occludens-1 (ZO-1) have also been demonstrated to be at the slit diaphragm [39]. 

The slit diaphragm contains transmembrane proteins such as nephrin and Neph1, which are unique to podocytes, as well as proteins such as Fat-1, P-cadherins, and catenins, which are typical of an adherence junction. 

Nephrin belongs to the immunoglobulin superfamily whose members are involved in cell-cell adhesion. Nephrin has an important role in maintaining the structure of the podocyte slit membrane, as shown by nephrin-deficient mice which develop proteinuria and foot process effacement [40]. Moreover, the injection of anti-nephrin antibody in animals also results in foot process effacement, and nephrin mutations were observed in patients with the congenital nephrotic syndrome of the Finnish type [41, 42]. 

It is thought that nephrin binds across the junction to itself or a similar protein called NEPH1 [43, 44]. This cross-junctional binding has been postulated to form a physical sieve that creates a size-selective pore in the slit diaphragm [45] (Figure 1).

Podocin (NPHS2, OMIM 604766), a member of the stomatin protein family, is exclusively expressed in the podocytes and localizes at the insertion of the slit diaphragm (Figure 1). This protein, like nephrin, associates with lipid rafts and recruits nephrin and CD2AP in these rafts ensuring a stable and proper functioning filtration barrier. Podocin dysfunction leads to alterations of the slit diaphragm assembly and to proteinuria in experimental models. NPHS2^−/−^ mice develop proteinuria and massive mesangial sclerosis with enlarged and focally vacuolized podocytes along with a rapid progression to sclerosis with aging [46]. In humans, podocin mutations are mainly associated with the autosomal recessive steroid-resistant nephrotic syndrome [47].

The transient receptor potential cation (TRPC) is a member of a family of proteins involved in the regulation of Ca^2+^ influx. These ion channels can be activated subsequently to either depletion of Ca^2+^ from internal stores or through receptor-mediated processes [48]. TRPC6 (OMIM 603652) is localized to the podocyte cell body, primary processes and in close vicinity to the slit membrane where it interacts with nephrin and podocin (not CD2AP). TRPC6 is also abundantly expressed in mesangial cells [49]. High glucose downregulates the TRPC6 protein, which might contribute to the impaired Ca^2+^ signaling of mesangial cells. This effect may explain the alteration in the mesangial contractile function due to a reduced Ca^2+^ influx observed in diabetic nephropathy [50]. 

TRPC6 was also found mutated in families with an autosomal dominant form of FSGS [51]. These mutations may cause a gain of function, and consequently an enhanced influx of Ca^2+^, especially after the activation of the G-protein-coupled receptor AT1 by angiotensin II (Ang II), this resulting in an altered channel regulation or an altered interaction with other slit diaphragm proteins like nephrin and podocin, which leads to proteinuria. In addition to the effects of gain-of-function mutations in the TRPC6 gene, also elevated levels of wild-type TRPC6 protein in some acquired glomerular diseases, like membranous nephropathy and puromycin aminonucleoside-induced albuminuria, may lead to podocyte dysfunction [52]. Recently it has been demonstrated that Ang II participates in the podocyte injury by increasing TRPC6 expression via an NFAT-mediated positive feedback signaling pathway. This finding highlights the crucial role of TRPC6 in the pathogenesis of podocyte injury and proteinuria [53].

Although, nephrin, podocin, VEGF, and synaptopodin are well-known podocyte markers, other proteins expressed in glomerular podocytes, such as glomerular epithelial protein 1 (GLEPP-1) and podoplanin, have also been involved in a number of glomerular diseases. Reduction in GLEPP-1 has been found associated to podocytopathies not only in biopsies of patients with IgA nephropathy and FSGS [54] but also in women with preeclampsia [55]. In addition, GLEPP-1 is considered a contributor to podocyte's foot process structure regulation. GLEPP-1 is a 132 kDa membrane protein tyrosine phosphatase with a large extracellular domain containing eight fibronectin-type-III-like repeats, a hydrophobic transmembrane segment, and a single protein-tyrosin phosphatase domain. Podoplanin, a 43 kDa integral membrane glycoprotein localized on the surface of rat podocytes, is downregulated in puromycin nephrosis [56]. Additionally, in the spontaneously proteinuric Dahl SS rat, segmental loss of podoplanin expression accompanied proteinuria and preceded widespread podocyte alterations for several weeks [57].

**The Mammalian Target of Rapamycin (mTOR) and Autophagy in Podocytes**

The mTOR is an evolutionarily conserved protein kinase [58]. The mTOR forms two distinct functional multiprotein kinase complexes, termed TOR complex 1 (TORC1) and TORC2 [59], which mutually phosphorylate different substrates and regulate a wide array of essential cellular processes including translation, transcription, and autophagy. mTOR is active in several types of cancer and plays a role in a variety of other serious human diseases, including diabetes, neurodegenerative disorders, and polycystic kidney disease.

Recent studies suggested that mTORC1 inhibition by rapamycin or everolimus can favourably modify glomerular diseases, such as minimal change disease [60], focal segmental glomerulosclerosis [61], membranous nephropathy [62, 63], crescentic glomerulonephritis [64], and diabetic nephropathy [65]. In diabetic animals rapamycin could prevent GBM thickening, glomerular hypertrophy, mesangial expansion, and renal macrophage [65]. Although the inhibition of mTORC1 activity attenuates pathological phenotypes of various different renal diseases in rodent models, rapamycin treatment has been associated with proteinuria in humans [66, 67]. Recent studies suggest that disruption of the autophagic pathway may play a role in the pathogenesis of proteinuria in patients treated with mTOR inhibitors [68].

Autophagy is an important homeostatic and quality control mechanism that maintains cellular integrity and has been shown to be essential for long-lived postmitotic cells, such as podocytes [69]. Autophagy refers to the process of self-degradation of cellular components in which proteins and organelles are sequestered and modified within cytosolic double-membrane vesicles, the autophagosomes, and subsequently delivered to the lysosome [70].

Although the mechanism by which mTOR regulates autophagy remains unclear, the induction of autophagy by mTORC1 inhibition is largely responsible for the potent effect of starvation on cell size, and rapamycin induces autophagy in a wide variety of cell types and species by inhibiting the activity of mTORC1 [71].

Whereas physiological level of mTOR activity inhibits autophagy in podocytes, mTOR reactivation allows autophagolysosomal reformation and the cycle of autophagy to complete itself. On the other hand, mTOR inhibition disrupts the autophagic pathway at two points. First, it relieves chronic suppression, resulting in activation and enhanced autophagy. Furthermore, mTOR inhibition will also lead to suppression of the reformation of lysosomes and autophagosomes, ultimately resulting in an accumulation of autolysosomal vesicle damaged intracellular organelles such as mitochondria and cell death [68].

Since autophagy has been recently identified as a crucial factor for glomerular maintenance and glomerular aging [69], it is very reasonable to assume that autophagy could be an interesting and novel therapeutic target for the treatment of glomerulopathies. However, in any attempt at manipulating podocyte autophagy therapeutically, it will be important to take into account the dynamic nature of the changes that occur in the autophagic system and related protein degradative pathways during the course of glomerular disease.

**APOL1 and Podocyte**

Recently, using the strategy of mapping by admixture linkage disequilibrium (MALD), two groups identified MYH9 as one of the genes underlying the ethnicity-driven health disparity in both end-stage renal disease (ESRD) [72] and FSGS [73].

Variation in MYH9 was estimated to account for about 70% of ESRD in non-diabetic African American patients [74]. Upon further examination, however, it was suggested that the true association with ESRD was with the apolipoprotein L-1 gene (APOL1), due to both the stronger statistical association with that gene and the lack of identification of causal functional variants in MYH9 [75–77].

Localization of APOL1 within the podocyte was demonstrated using immunofluorescent confocal microscopy to colocalize APOL1 with markers for podocytes (GLEPP1 and synaptopodin) in normal human kidney sections [78].

Notably, in renal biopsies of human FSGS and HIVAN, diminution in podocyte APOL1 expression preceded decreases in GLEPP1 and synaptopodin [78]. APOL1 appears to be constitutively. Some reports suggest that APOL1 sequesters phosphatidic acid and cardiolipin and promotes autophagocytic cell death [79]. Due to the importance of autophagy in modulation of podocyte aging [80], APOL1 may have substantial roles in podocyte homeostasis and survival. Importantly, APOL1 expression by cultured podocytes could also be induced by inflammatory mediators, such as tumor necrosis factor-*α* and lipopolysaccharide, indicating potential modulation by systemic influences [78].

Future studies focusing the clinical implications of APOL1 genotype in the setting of glomerulopathies and hypertension-associated kidney disease as well as exploring the cellular and molecular mechanisms of APOL1-associated disease may help to clarify these recent findings thus leading to new treatment approaches.

### 2.4. Podocytes and Vasoactive Molecules

In addition to forming a molecular glomerular filtration barrier, podocytes modulate filtration surface by counteracting the intracapillary pressure. The latter property is associated with a well-developed system of contractile proteins expressed in these cells as already mentioned above. Furthermore, podocytes not only express receptors for a number of vasoactive factors including atrial natriuretic peptide (ANP), nitric oxide (NO), and Ang II but are also capable of producing some of these hormones [81]. Additionally, podocytes present a number of second messenger systems including cyclic GMP and phospholipase C/inositol 1,4,5-triphosphate systems [82]. All these strongly suggest that at least some of the podocyte functions may be regulated by vasoactive substances. 

Some experiments indicate that Ang II interacts with the systems generating vasoactive factors, either enhancing or antagonizing their activity [83–85]. Additionally, the modulatory effects of Ang II on the NO-dependent and ANP-dependent cGMP generation have also been reported [86–88]. In podocytes, Ang II has been shown to inhibit ANP-stimulated production of cGMP [89]. 

**Figure 3:**
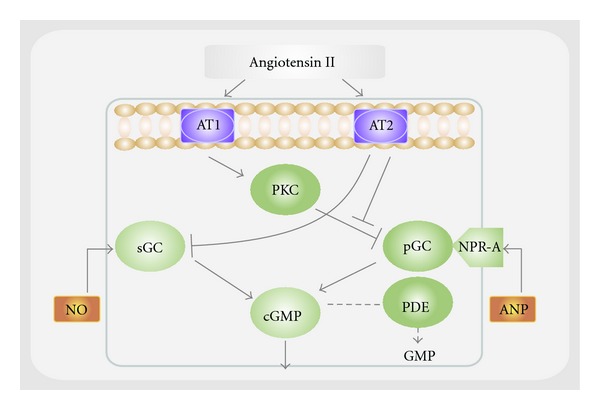
Schematic representation of the interaction between angiotensin II and guanylyl cyclase-dependent systems through AT1 and AT2 receptor in the podocyte. In podocytes, the guanylyl cyclase system may be modulated by angiotensin II via both AT1 receptor through pGC or AT2 receptor through sGC. In the absence of angiotensin II, both ANP and NO may also activate pGC and sGC, respectively. Abbreviations: cGMP: cyclic guanosine 5′-monophosphate; ANP: atrial natriuretic peptide; NO: nitric oxide; NPR-A: guanylyl cyclase-linked receptor for ANP; PKC: protein kinase C; sGC: soluble guanylyl cyclase; pGC: particulate guanylyl cyclase; PDE: phosphodiesterase. (Adapted from [81]).

The synthesis of cGMP in cultured podocytes is modulated by Ang II via AT1 or AT2 receptors [82] (Figure 3). 

### 2.5. Is the Glomerular Filtration Barrier Actually Efficient?

How much protein crosses the glomerular barrier? Or in other words, how permeable is the glomerular barrier really? Undoubtedly, this last question has been a theme of major controversy for a long time. However, it is worth mentioning that most of the available information supports the idea that, in physiological conditions, the glomerular barrier is a functional structure with size and charge selectivity [90]. In contrast to this concept, some few studies [91, 92] postulate a glomerular sieving coefficient (GSC) for albumin of 0.034, which is much higher than the originally reported one (0.0006) [90]. Moreover, these experiments describe that renal albumin filtration in nonproteinuric rats is several times greater than previously measured filtrations and is followed by rapid endocytosis into proximal tubule cells. These findings suggest that the glomerular filter normally leaks albumin at nephrotic levels and that dysfunction of this retrieval pathway leads to proteinuria. Nevertheless, this hypothesis has been severely questioned on the light of the studies on megalin-cubilin, in which there is no retrieval of intact albumin. Filtered albumin binds to the megalin-cubilin complex of proximal tubule cells and is internalized, degraded, and released to blood as amino acids [93, 94]. Additionally, methodological objections to this so-called albumin retrieval hypothesis were also done after following physiological experiments using different techniques [95, 96]. Consequently, only modest amounts of albumin normally cross the barrier, but the amounts can increase greatly if there are barrier defects. 

### 2.6. Proteinuria and Tubulointerstitial Response

In the last years, there has been an extensive debate on whether tubular albumin reabsorption and the subsequent lysosomal degradation may in fact result in tubular injury due to protein overload. The role of tubular reabsorption in urine protein homeostasis seems to be critical for the development of CKD [97]. During normal physiological conditions, all filtered proteins are efficiently internalized by the receptor complex megalin/cubilin/amnionless (AMN) by the proximal tubular epithelial cells, thus resulting in virtually protein-devoid urine [98]. In the proximal tubular cell, the proteins are degraded in lysosomes and substances such as vitamins are transported basally for reuse. However, in the case of glomerular injury, filtration of low-molecular-weight proteins increases and larger proteins start to penetrate the glomerular filtration barrier. Then, cells in the proximal tubule are thereby exposed to more, and new, proteins that overload the receptor-binding sites, thus leading to proteinuria. Furthermore, in the tubular cell, lysosomal degradation is unable to handle the increased amount of internalized protein, resulting in protein-clotted lysosomes. Although in most instances the primary event in triggering renal damage is glomerular injury, it is widely accepted that the severity in tubular and interstitial alterations is the driver for the development of fibrotic lesions, eventually resulting in ESRD. 

**Figure 4:**
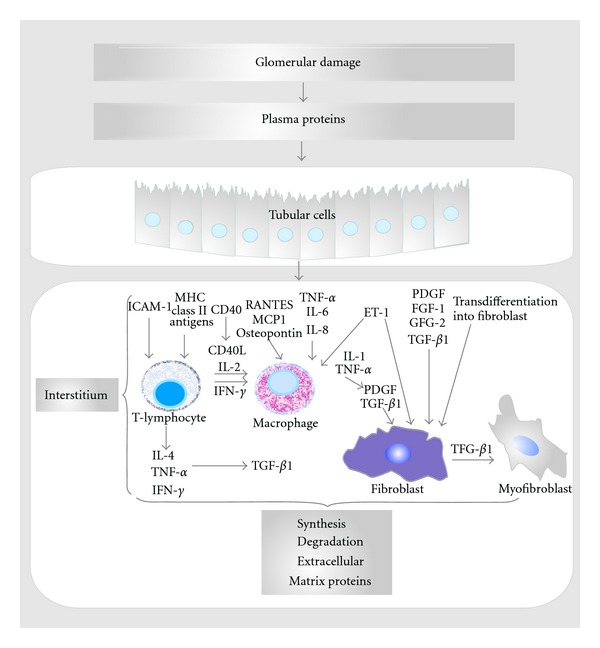
Molecules involved in potential mechanisms in the development of proteinuria-induced renal tubulointerstitial injury. The tubulointerstitial damage induced by persistent proteinuria in glomerular diseases is due to the concomitant injuring effects of multiple cellular and biochemical events. Specific proteins have been shown to stimulate production of cytokines, chemotactants, and matrix proteins by tubular epithelial cells, which may stimulate interstitial inflammation and scarring. Abbreviations: MHC: major histocompatibility complex; ICAM-1: intercellular adhesion molecule 1; MCP1: monocyte chemotactic protein-1; TNF-*α*: tumor necrosis factor-alpha; FGF: fibroblast growth factor; TGF-*β*1: transforming growth factor beta; PDGF: platelet-derived growth factor; ET-1: endothelin; RANTES: regulated upon activation, normal T-cell expressed and secreted; ECM: extracellular matrix. (Adapted from [99]).

Recent research has focused on how glomerular injury spreads to the tubulointerstitium, and currently, four possible mechanisms are being discussed: (1) obstruction of the urinary pole, (2) proteinuria-induced overload of the proximal tubule, (3) chronic hypoxia, and (4) inflammation induced by a glomerulotubular feedback loop. Figure 4 shows the diverse molecules involved in potential mechanisms in the development of renal tubulointestitial injury induced by proteinuria [99].

**Therapeutic Approaches in Managing Patients with Proteinuria**

It is widely accepted that the podocyte is the target of many types of injury mechanisms regardless of their nature. Hence, the loss of podocytes contributes to the development of glomerulosclerosis. Among them, the insult against podocyte membrane antigens as in membranous nephropathy and minimal change disease and the consequence of hemodynamic injury produced by a reduced nephron number are both relevant causes linked to podocyte damage. Moreover, the already mentioned mutations in the genes of the glomerular filtration barrier-participating proteins (nephrin, laminin, TRPC6, *α*-actinin-4, CD2AP, type IV collagen, etc.) as well as the tremendous impact of metabolic disarrangements (especially diabetes and dyslipidemias) are associated with podocyte lesions [100–103]. Some other etiologies like protein overload states, drugs/toxins (NSADs, adriamycin), infectious diseases, and still unknown causes including idiopathic FSGS are also recognized causes of damage in glomerular visceral epithelial cell.

Undoubtedly, the inhibition of the renin-angiotensin-aldosterone system (RAAS) is associated with a maximum reduction in proteinuria and long-term renal risk reduction as well as a long-term renoprotection. Numerous clinical and experimental studies have demonstrated to reduce or control proteinuria by using either an angiotensin-converting enzyme inhibitor (ACEI) or angiotensin II receptor blocker (ARB) as a single therapy or in a dual blockade of RAAS [104–108]. However, due to individual risk factors, some patients present variable response to these therapeutic agents. It is worth emphasizing that dietary sodium restriction and diuretic therapy are similarly effective in improving the antiproteinuric action of the inhibition of RAAS. Therefore, the combination of both low-sodium diet and diuretic therapy may result in the highest reduction in proteinuria. Recently, direct renin inhibition has been included within the broad spectrum of RAAS blockade drugs. Aliskiren, a new direct renin inhibitor, reduced albuminuria significantly as it was demonstrated in a large study, in which proteinuric patients at the top of losartan treatment achieved a better control of albuminuria [109]. The blockade of aldosterone by either spironolactone or eplerenone together with and ACEI or ARB has been shown to have beneficial effects in patients with proteinuria, although the potential risk of hyperkalemia is increased [110, 111].

Although the interaction against the RAAS is the current therapeutic first line in patients with proteinuria, other drugs that may lower proteinuria independent of the RAAS action have become of great interest. In that sense and considering some pathophysiological mechanisms, new strategies such as vitamin D receptor activators and monocyte chemotactic protein-1 antagonists as well as endothelin antagonists emerge as potential alternatives in this setting. In addition, the combined therapy of these agents with the RAAS inhibition may potentiate antiproteinuric effects, thus exerting further renal protection.

In the field of new therapeutic perspectives in controlling glomerular damage, the VEGF antagonism emerges as a potential alternative. Based on the preliminary experimental studies in streptozotocin-diabetic rats with antibodies against VEGF in which renal function was improved [112], VEGF antagonism has been postulated as a promising tool in the treatment of diabetic nephropathy. Reinforcing this concept, nephropathy in db/db mice was attenuated by a small molecular inhibitor of angiogenesis [113] and by VEGF tyrosine kinase inhibitor [114]. However, tilting the balance against VEGF may cause harm. Chronic VEGF suppression may worsen interstitial fibrosis, and anti-VEGF antibody treatment was related to an exaggerated cystic response of the proximal tubules in cystic rats and severe kidney injury that was associated with low renal VEGF and high HIF-1*α* levels [115, 116]. Consequently, an extensive bulk of work is still needed to confidently assess the actually beneficial mechanisms and safety of VEGF antagonism for proteinuria. At present, anti-VEGF therapy remains as a double-edged sword in the nephrology scenario.

Taking into account the importance of the glomerular filtration barrier integrity, a more suitable therapeutic approach in order to stop or, at least, achieve a substantial reduction in proteinuria remains a big clinical challenge.

## References

[1] Bello AK, Hemmelgarn B, Lloyd A (2011). Associations among estimated glomerular filtration rate, proteinuria, and adverse cardiovascular outcomes. *Clinical Journal of the American Society of Nephrology*.

[2] de Jong PE, Gansevoort RT, Bakker SJL (2007). Macroalbuminuria and microalbuminuria: do both predict renal and cardiovascular events with similar strength?. *Journal of Nephrology*.

[3] Levey AS, Cattran D, Friedman A (2009). Proteinuria as a surrogate outcome in CKD: report of a scientific workshop sponsored by the National Kidney Foundation and the US Food and Drug Administration. *American Journal of Kidney Diseases*.

[4] Hemmelgarn BR, Manns BJ, Lloyd A (2010). Relation between kidney function, proteinuria, and adverse outcomes. *The Journal of the American Medical Association*.

[5] Hsu CY, Chertow GM (2000). Chronic renal confusion: insufficiency, failure, dysfunction, or disease. *American Journal of Kidney Diseases*.

[6] Hillege HL, Fidler V, Diercks GFH (2002). Urinary albumin excretion predicts cardiovascular and noncardiovascular mortality in general population. *Circulation*.

[7] Klausen K, Borch-Johnsen K, Feldt-Rasmussen B (2004). Very low levels of microalbuminuria are associated with increased risk of coronary heart disease and death independently of renal function, hypertension, and diabetes. *Circulation*.

[8] Mann JFE, Gerstein HC, Poque J, Bosch J, Yusuf S (2001). Renal insufficiency as a predictor of cardiovascular outcomes and the impact of ramipril: the HOPE randomized trial. *Annals of Internal Medicine*.

[9] Viswanathan G, Upadhyay A (2011). Assessment of proteinuria. *Advances in Chronic Kidney Disease*.

[10] Mundel P, Reiser J (2010). Proteinuria: an enzymatic disease of the podocyte. *Kidney International*.

[11] Pries AR, Secomb TW, Gaehtgens P (2000). The endothelial surface layer. *Pflugers Archiv European Journal of Physiology*.

[12] Drumond MC, Deen WM (1994). Structural determinants of glomerular hydraulic permeability. *American Journal of Physiology*.

[13] Jeansson M, Bjorck K, Tenstad O, Haraldsson B (2009). Adriamycin alters glomerular endothelium to induce proteinuria. *Journal of the American Society of Nephrology*.

[14] Jeansson M, Haraldsson B (2006). Morphological and functional evidence for an important role of the endothelial cell glycocalyx in the glomerular barrier. *American Journal of Physiology*.

[15] Eremina V, Baelde HJ, Quaggin SE (2007). Role of the VEGF-A signaling pathway in the glomerulus: evidence for crosstalk between components of the glomerular filtration barrier. *Nephron—Physiology*.

[16] Eremina V, Jefferson JA, Kowalewska J (2008). VEGF inhibition and renal thrombotic microangiopathy. *The New England Journal of Medicine*.

[17] Satchell SC, Braet F (2009). Glomerular endothelial cell fenestrations: an integral component of the glomerular filtration barrier. *American Journal of Physiology*.

[18] Fan Q, Xing Y, Ding J, Guan N (2009). Reduction in VEGF protein and phosphorylated nephrin associated with proteinuria in adriamycin nephropathy rats. *Nephron—Experimental Nephrology*.

[19] Fan L, Wakayama T, Yokoyama S, Amano O, Iseki S (2002). Downregulation of vascular endothelial growth factor and its receptors in the kidney in rats with puromycin aminonucleoside nephrosis. *Nephron*.

[20] Horita Y, Miyazaki M, Koji T (1998). Expression of vascular endothelial growth factor and its receptors in rats with protein-overload nephrosis. *Nephrology Dialysis Transplantation*.

[21] Bailey E, Bottomley MJ, Westwell S (1999). Vascular endothelial growth factor mRNA expression in minimal change, membranous, and diabetic nephropathy demonstrated by non-isotopic in situ hybridisation. *Journal of Clinical Pathology*.

[22] Matsumoto K, Kanmatsuse K (2001). Elevated vascular endothelial growth factor levels in the urine of patients with minimal-change nephrotic syndrome. *Clinical Nephrology*.

[23] Hohenstein B, Colin M, Foellmer C (2010). Autocrine VEGF-VEGF-R loop on podocytes during glomerulonephritis in humans. *Nephrology Dialysis Transplantation*.

[24] Jefferson JA, Nelson PJ, Najafian B, Shankland SJ (2011). Podocyte disorders: core curriculum 2011. *American Journal of Kidney Diseases*.

[25] Miner JH (2011). Glomerular basement membrane composition and the filtration barrier. *Pediatric Nephrology*.

[26] Hudson BG, Tryggvason K, Sundaramoorthy M, Neilson EG (2003). Alport’s syndrome, Goodpasture’s syndrome, and type IV collagen. *The New England Journal of Medicine*.

[27] Cosgrove D, Meehan DT, Grunkemeyer JA (1996). Collagen COL4A3 knockout: a mouse model for autosomal Alport syndrome. *Genes and Development*.

[28] van den Hoven MJ, Wijnhoven TJ, Li JP (2008). Reduction of anionic sites in the glomerular basement membrane by heparanase does not lead to proteinuria. *Kidney International*.

[29] Zenker M, Pierson M, Jonveaux P, Reis A (2005). Demonstration of two novel LAMB2 mutations in the original Pierson syndrome family reported 42 years ago. *American Journal of Medical Genetics Part A*.

[30] Sachs N, Kreft M, van den Bergh Weerman MA (2006). Kidney failure in mice lacking the tetraspanin CD151. *Journal of Cell Biology*.

[31] Dai C, Stolz DB, Bastacky SI (2006). Essential role of integrin-linked kinase in podocyte biology: bridging the integrin and slit diaphragm signaling. *Journal of the American Society of Nephrology*.

[32] El-Aouni C, Herbach N, Blattner SM (2006). Podocyte-specific deletion of integrin-linked kinase results in severe glomerular basement membrane alterations and progressive glomerulosclerosis. *Journal of the American Society of Nephrology*.

[33] Kang YS, Li Y, Dai C, Kiss LP, Wu C, Liu Y (2010). Inhibition of integrin-linked kinase blocks podocyte epithelial-mesenchymal transition and ameliorates proteinuria. *Kidney International*.

[34] McCarthy HJ, Saleem MA (2010). Genetics in clinical practice: nephrotic and proteinuric syndromes. *Nephron—Experimental Nephrology*.

[35] Nielsen JS, McNagny KM (2009). The role of podocalyxin in health and disease. *Journal of the American Society of Nephrology*.

[36] Kurihara H, Anderson JM, Kerjaschki D, Farquhar MG (1992). The altered glomerular filtration slits seen in puromycin aminonucleoside nephrosis and protamine sulfate-treated rats contain the tight junction protein ZO-1. *American Journal of Pathology*.

[37] Torres-Rasgado E, Fouret G, Carbonneau MA, Leger CL (2007). Peroxynitrite mild nitration of albumin and LDL-albumin complex naturally present in plasma and tyrosine nitration rate-albumin impairs LDL nitration. *Free Radical Research*.

[38] Löwik MM, Groenen PJTA, Pronk I (2007). Focal segmental glomerulosclerosis in a patient homozygous for a CD2AP mutation. *Kidney International*.

[39] Fukasawa H, Bornheimer S, Kudlicka K, Farquhar MG (2009). Slit diaphragms contain tight junction proteins. *Journal of the American Society of Nephrology*.

[40] Putaala H, Soininen R, Kilpelainen P, Wartiovaara J, Tryggvason K (2001). The murine nephrin gene is specifically expressed in kidney, brain and pancreas: inactivation of the gene leads to massive proteinuria and neonatal death. *Human Molecular Genetics*.

[41] Orikasa M, Matsui K, Oite T, Shimizu F (1988). Massive proteinuria induced in rats by a single intravenous injection of a monoclonal antibody. *Journal of Immunology*.

[42] Khoshnoodi J, Sigmundsson K, Ofverstedt LG (2003). Nephrin promotes cell-cell adhesion through homophilic interactions. *American Journal of Pathology*.

[43] Santín S, García-Maset R, Ruíz P (2009). Nephrin mutations cause childhood-and adult-onset focal segmental glomerulosclerosis. *Kidney International*.

[44] Barletta GM, Kovari IA, Verma RK, Kerjaschki D, Holzman LB (2003). Nephrin and Neph1 co-localize at the podocyte foot process intercellular junction and form cis hetero-oligomers. *The Journal of Biological Chemistry*.

[45] Wartiovaara J, Ofverstedt LG, Khoshnoodi J (2004). Nephrin strands contribute to a porous slit diaphragm scaffold as revealed by electron tomography. *Journal of Clinical Investigation*.

[46] Roselli S, Heidet L, Sich M (2004). Early glomerular filtration defect and severe renal disease in podocin-deficient mice. *Molecular and Cellular Biology*.

[47] Sako M, Nakanishi K, Obana M (2005). Analysis of NPHS1, NPHS2, ACTN4, and WT1 in Japanese patients with congenital nephrotic syndrome. *Kidney International*.

[48] Riccio A, Medhurst AD, Mattei C (2002). mRNA distribution analysis of human TRPC family in CNS and peripheral tissues. *Molecular Brain Research*.

[49] Sours S, Du J, Chu S, Ding M, Zhou XJ, Ma R (2006). Expression of canonical transient receptor potential (TRPC) proteins in human glomerular mesangial cells. *American Journal of Physiology*.

[50] Graham S, Ding M, Sours-Brothers S, Yorio T, Ma JX, Ma R (2007). Downregulation of TRPC6 protein expression by high glucose, a possible mechanism for the impaired Ca^2+^ signaling in glomerular mesangial cells in diabetes. *American Journal of Physiology*.

[51] Winn MP, Conlon PJ, Lynn KL (2005). Medicine: a mutation in the TRPC6 cation channel causes familial focal segmental glomerulosclerosis. *Science*.

[52] Moller CC, Wei C, Altintas MM (2007). Induction of TRPC6 channel in acquired forms of proteinuric kidney disease. *Journal of the American Society of Nephrology*.

[53] Nijenhuis T, Sloan AJ, Hoenderop JGJ (2011). Angiotensin II contributes to podocyte injury by increasing TRPC6 expression via an NFAT-mediated positive feedback signaling pathway. *American Journal of Pathology*.

[54] Hill GS, Karoui KE, Karras A (2011). Focal segmental glomerulosclerosis plays a major role in the progression of IgA nephropathy. I. Immunohistochemical studies. *Kidney International*.

[55] Zhao S, Gu X, Groome LJ, Wang Y (2009). Decreased nephrin and GLEPP-1, but increased VEGF, Flt-1, and nitrotyrosine, expressions in kidney tissue sections from women with preeclampsia. *Reproductive Sciences*.

[56] Breiteneder-Geleff S, Matsui K, Soleiman A (1997). Podoplanin, novel 43-kd membrane protein of glomerular epithelial cells, is down-regulated in puromycin nephrosis. *American Journal of Pathology*.

[57] Koop K, Eikmans M, Wehland M (2008). Selective loss of podoplanin protein expression accompanies proteinuria and precedes alterations in podocyte morphology in a spontaneous proteinuric rat model. *American Journal of Pathology*.

[58] Inoki K, Huber TB (2012). Mammalian target of rapamycin signaling in the podocyte. *Current Opinion in Nephrology and Hypertension*.

[59] Loewith R, Jacinto E, Wullschleger S (2002). Two TOR complexes, only one of which is rapamycin sensitive, have distinct roles in cell growth control. *Molecular Cell*.

[60] Ito N, Nishibori Y, Ito Y (2011). MTORC1 activation triggers the unfolded protein response in podocytes and leads to nephrotic syndrome. *Laboratory Investigation*.

[61] Rangan GK, Coombes JD (2007). Renoprotective effects of sirolimus in non-immune initiated focal segmental glomerulosclerosis. *Nephrology Dialysis Transplantation*.

[62] Bonegio RGB, Fuhro R, Wang Z (2005). Rapamycin ameliorates proteinuria-associated tubulointerstitial inflammation and fibrosis in experimental membranous nephropathy. *Journal of the American Society of Nephrology*.

[63] Naumovic R, Jovovic D, Basta-Jovanovic G (2007). Effects of rapamycin on active Heymann nephritis. *American Journal of Nephrology*.

[64] Kurayama R, Ito N, Nishibori Y (2011). Role of amino acid transporter LAT2 in the activation of mTORC1 pathway and the pathogenesis of crescentic glomerulonephritis. *Laboratory Investigation*.

[65] Yang Y, Wang J, Qin L (2007). Rapamycin prevents early steps of the development of diabetic nephropathy in rats. *American Journal of Nephrology*.

[66] Amer H, Cosio FG (2009). Significance and management of proteinuria in kidney transplant recipients. *Journal of the American Society of Nephrology*.

[67] Torras J, Herrero-Fresneda I, Gulias O (2009). Rapamycin has dual opposing effects on proteinuric experimental nephropathies: is it a matter of podocyte damage. *Nephrology Dialysis Transplantation*.

[68] Cinà DP, Onay T, Paltoo A (2012). Inhibition of MTOR disrupts autophagic flux in podocytes. *Journal of the American Society of Nephrology*.

[69] Hartleben B, Gödel M, Meyer-Schwesinger C (2010). Autophagy influences glomerular disease susceptibility and maintains podocyte homeostasis in aging mice. *Journal of Clinical Investigation*.

[70] Mizushima N, Levine B, Cuervo AM, Klionsky DJ (2008). Autophagy fights disease through cellular self-digestion. *Nature*.

[71] Neufeld TP (2010). TOR-dependent control of autophagy: biting the hand that feeds. *Current Opinion in Cell Biology*.

[72] Kao WHL, Klag MJ, Meoni LA (2008). MYH9 is associated with nondiabetic end-stage renal disease in African Americans. *Nature Genetics*.

[73] Kopp JB, Smith MW, Nelson GW (2008). MYH9 is a major-effect risk gene for focal segmental glomerulosclerosis. *Nature Genetics*.

[74] Bostrom MA, Freedman BI (2010). The spectrum of MYH9-associated nephropathy. *Clinical Journal of the American Society of Nephrology*.

[75] Genovese G, Friedman DJ, Ross MD (2010). Association of trypanolytic ApoL1 variants with kidney disease in African Americans. *Science*.

[76] Genovese G, Tonna SJ, Knob AU (2010). A risk allele for focal segmental glomerulosclerosis in African Americans is located within a region containing APOL1 and MYH9. *Kidney International*.

[77] Tzur S, Rosset S, Shemer R (2010). Missense mutations in the APOL1 gene are highly associated with end stage kidney disease risk previously attributed to the MYH9 gene. *Human Genetics*.

[78] Madhavan SM, O’Toole JF, Konieczkowski M, Ganesan S, Bruggeman LA, Sedor JR (2011). APOL1 localization in normal kidney and nondiabetic kidney disease. *Journal of the American Society of Nephrology*.

[79] Zhaorigetu S, Wan G, Kaini R, Jiang Z, Hu CAA (2008). ApoL1, a BH3-only lipid-binding protein, induces autophagic cell death. *Autophagy*.

[80]  Weide T, Huber TB (2011). Implications of autophagy for glomerular aging and disease. *Cell Tissue Research *.

[81] Lewko B, Gołos M, Latawiec E, Angielski S, Stepinski J (2006). Regulation of cGMP synthesis in cultured podocytes by vasoactive hormones. *Journal of Physiology and Pharmacology*.

[82] Pavenstadt H, Kriz W, Kretzler M (2003). Cell biology of the glomerular podocyte. *Physiological Reviews*.

[83] Patzak A, Lai E, Persson PB, Persson AEG (2005). Angiotensin II-nitric oxide interaction in glomerular arterioles. *Clinical and Experimental Pharmacology and Physiology*.

[84] Gauquelin G, Schiffrin EL, Garcia R (1991). Downregulation of glomerular and vascular atrial natriuretic factor receptor subtypes by angiotensin II. *Journal of Hypertension*.

[85] Nakayama I, Kawahara Y, Tsuda T, Okuda M, Yokoyama M (1994). Angiotensin II inhibits cytokine-stimulated inducible nitric oxide synthase expression in vascular smooth muscle cells. *The Journal of Biological Chemistry*.

[86] Zhang C, Mayeux PR (1998). Angiotensin II signaling activates the NO-cGMP pathway in rat propximal tubules. *Life Sciences*.

[87] Haneda M, Kikkawa R, Maeda S (1991). Dual mechanism of angiotensin II inhibits ANP-induced mesangial cGMP accumulation. *Kidney International*.

[88] Kim D, Aizawa T, Wei H (2005). Angiotensin II increases phosphodiesterase 5A expression in vascular smooth muscle cells: a mechanism by which angiotensin II antagonizes cGMP signaling. *Journal of Molecular and Cellular Cardiology*.

[89] Golos M, Lewko B, Bryl E (2002). Effect of angiotensin II on ANP-dependent guanylyl cyclase activity in cultured mouse and rat podocytes. *Kidney and Blood Pressure Research*.

[90] Tojo A, Endou H (1992). Intrarenal handling of proteins in rats using fractional micropuncture technique. *American Journal of Physiology*.

[91] Russo LM, Sandoval RM, McKee M (2007). The normal kidney filters nephrotic levels of albumin retrieved by proximal tubule cells: retrieval is disrupted in nephrotic states. *Kidney International*.

[92] Sandoval RM, Wagner MC, Patel M (2012). Multiple factors influence glomerular albumin permeability in rats. *Journal of the American Society of Nephrology*.

[93] Birn H, Christensen EI (2006). Renal albumin absorption in physiology and pathology. *Kidney International*.

[94] Park CH, Maack T (1984). Albumin absorption and catabolism by isolated perfused proximal convoluted tubules of the rabbit. *Journal of Clinical Investigation*.

[95] Tanner GA (2009). Glomerular sieving coefficient of serum albumin in the rat: a two-photon microscopy study. *American Journal of Physiology*.

[96] Comper WD, Haraldsson B, Deen WM (2008). Resolved: normal glomeruli filter nephrotic levels of albumin. *Journal of the American Society of Nephrology*.

[97] Theilig F, Kriz W, Jerichow T (2007). Abrogation of protein uptake through megalin-deficient proximal tubules does not safeguard against tubulointerstitial injury. *Journal of the American Society of Nephrology*.

[98] Nielsen R, Christensen EI (2010). Proteinuria and events beyond the slit. *Pediatric Nephrology*.

[99] D’Amico G (1999). Tubulointerstitium as predictor of progression of glomerular diseases. *Nephron*.

[100] Pollak MR (2002). Inherited podocytopathies: FSGS and nephrotic syndrome from a genetic viewpoint. *Journal of the American Society of Nephrology*.

[101] Joles JA, Kunter U, Janssen U (2000). Early mechanisms of renal injury in hypercholesterolemic or hypertriglyceridemic rats. *Journal of the American Society of Nephrology*.

[102] Lee HS (2011). Mechanisms and consequences of hypertriglyceridemia and cellular lipid accumulation in chronic kidney disease and metabolic syndrome. *Histology and Histopathology*.

[103] Ziyadeh FN, Wolf G (2008). Pathogenesis of the podocytopathy and proteinuria in diabetic glomerulopathy. *Current Diabetes Reviews*.

[104] Ruggenenti P, Perna A, Gherardi G (1999). Renoprotective properties of ACE-inhibition in non-diabetic nephropathies with non-nephrotic proteinuria. *The Lancet*.

[105] Lewis EJ, Hunsicker LG, Clarke WR (2001). Renoprotective effect of the angiotensin-receptor antagonist irbesartan in patients with nephropathy due to type 2 diabetes. *The New England Journal of Medicine*.

[106] Brenner BM, Cooper ME, de Zeeuw D (2001). Effects of losartan on renal and cardiovascular outcomes in patients with type 2 diabetes and nephropathy. *The New England Journal of Medicine*.

[107] Maschio G, Alberti D, Janin G (1996). Effect of the angiotensin-converting-enzyme inhibitor benazepril on the progression of chronic renal insufficiency. *The New England Journal of Medicine*.

[108] Toblli JE, DeRosa G, Cao G, Piorno P, Pagano P (2004). ACE inhibitor and angiotensin type I receptor antagonist in combination reduce renal damage in obese Zucker rats. *Kidney International*.

[109] Parving HH, Persson F, Lewis JB, Lewis EJ, Hollenberg NK (2008). Aliskiren combined with losartan in type 2 diabetes and nephropathy. *The New England Journal of Medicine*.

[110] Mehdi UF, Adams-Huet B, Raskin P, Vega GL, Toto RD (2009). Addition of angiotensin receptor blockade or mineralocorticoid antagonism to maximal angiotensin-converting enzyme inhibition in diabetic nephropathy. *Journal of the American Society of Nephrology*.

[111] Epstein M, Williams GH, Weinberger M (2006). Selective aldosterone blockade with eplerenone reduces albuminuria in patients with type 2 diabetes. *Clinical Journal of the American Society of Nephrology*.

[112] de Vriese AS, Tilton RG, Elger M, Stephan CC, Kriz W, Lameire NH (2001). Antibodies against vascular endothelial growth factor improve early renal dysfunction in experimental diabetes. *Journal of the American Society of Nephrology*.

[113] Ichinose K, Maeshima Y, Yamamoto Y (2006). 2-(8-Hydroxy-6-methoxy-1-oxo-1H-2-benzopyran-3-yl) propionic acid, an inhibitor of angiogenesis, ameliorates renal alterations in obese type 2 diabetic mice. *Diabetes*.

[114] Sung SH, Ziyadeh FN, Wang A, Pyagay PE, Kanwar YS, Chen S (2006). Blockade of vascular endothelial growth factor signaling ameliorates diabetic albuminuria in mice. *Journal of the American Society of Nephrology*.

[115] Raina S, Honer M, Krämer SD (2011). Anti-VEGF antibody treatment accelerates polycystic kidney disease. *American Journal of Physiology*.

[116] Masuda Y, Shimizu A, Kataoka M (2010). Inhibition of capillary repair in proliferative glomerulonephritis results in persistent glomerular inflammation with glomerular sclerosis. *Laboratory Investigation*.

